# Unraveling the Links between Chronic Inflammation, Autoimmunity, and Spontaneous Cervicocranial Arterial Dissection

**DOI:** 10.3390/jcm12155132

**Published:** 2023-08-05

**Authors:** Hao Li, Shiyao Xu, Beibei Xu, Yutong Zhang, Jun Yin, Yi Yang

**Affiliations:** 1 Department of Neurology, The First Affiliated Hospital of Soochow University, Suzhou 215006, China; 20204032009@stu.suda.edu.cn (H.L.); 20214232026@stu.suda.edu.cn (B.X.); 2 The Institute of Neuroscience, Soochow University, Suzhou 215006, China; 20214254020@stu.suda.edu.cn (S.X.); 1830805068@stu.suda.edu.cn (Y.Z.); 3 Department of General Surgery, The First Affiliated Hospital of Soochow University, Suzhou 215006, China

**Keywords:** cervicocranial arterial dissection, autoimmune, inflammatory

## Abstract

Advances in imaging techniques have led to a rise in the diagnosis of spontaneous cervicocranial arterial dissection (SCCAD), which is now considered a common cause of stroke in young adults. However, our understanding of the pathophysiological mechanisms underlying SCCAD remains limited. Prior studies have proposed various factors contributing to arterial wall weakness or stress as potential causes for SCCAD. A combination of biopsies, case reports, and case–control studies suggests that inflammatory changes and autoimmunity may play roles in the cascade of events leading to SCCAD. In this review, we examine the close relationship between SCCAD, chronic inflammation, and autoimmune diseases, aiming to explore potential underlying pathophysiological mechanisms connecting these conditions. While some relevant hypotheses and studies exist, direct evidence on this topic is still relatively scarce. Further investigation of the underlying mechanisms in larger clinical cohorts is needed, and the exploration of animal models may provide novel insights.

## 1. Introduction

Cervicocranial arterial dissection (CCAD) refers to a hematoma within the wall of the cervical or intracranial artery. Based on etiology, CCAD is typically classified as spontaneous or traumatic [1]. The non-specific symptoms of spontaneous CCAD (SCCAD) depend on the involved artery, often leading to diagnostic challenges and delays [2,3]. As imaging techniques have advanced, SCCAD diagnosis has increased, and it is now recognized as a common cause of stroke in young adults [4,5]. Despite this, the pathophysiology and pathogenesis of SCCAD remain unclear. Researchers speculate that this condition may be multifactorial [6], with reported risk factors including minor trauma, hypertension, genetic susceptibility, pregnancy, postpartum, recent infection or inflammation, connective tissue abnormalities, and autoimmune diseases [7,8,9]. Intriguingly, epidemiological observations over the past decades suggest potential correlations between inflammation, tissue abnormalities, and SCCAD [10,11,12,13]. Recent data further emphasize shared biological mechanisms and risk factors between autoimmunity and SCCAD [14,15]. This review highlights the association between SCCAD and autoimmune diseases, providing epidemiological evidence to support possible underlying pathophysiological mechanisms between these two conditions. 

## 2. Data Acquisition

We conducted a comprehensive search of PubMed and EMBASE databases using the following keywords: “Cervicocranial arterial dissection”, “Cervical artery dissection”, “Dissection”, “Carotid dissection”, and “Vertebral dissection”, for articles published between 1 January 1992 and 1 December 2022. We identified evidence-based peer-reviewed articles, including randomized trials, case reports, and reviews. Our search was restricted to studies published in English or Chinese languages. 

Exclusion criteria: (1) duplicate case reports; (2) studies involving pediatric patients; (3) studies focusing on trauma and surgically induced CCAD; (4) studies lacking detailed methodology; (5) studies that solely examined intracranial hemorrhage associated with CCAD; (6) studies including patients not specifically identified as having CCAD; (7) non-peer-reviewed publications.

The authors conducted an initial screening of titles and abstracts, followed by a second screening in which the full text of selected articles was reviewed. Specifically for case–control studies and cohorts, we conducted a targeted screening of studies published prior to January 2000. This step was taken to ensure that our findings align with the current standards of care. The flow chart depicting the screening process is presented in Supplementary Materials.

## 3. Epidemiology of SCCAD

In Iran, a separate study suggested a crude incidence rate of about 1.20 cases per 100,000 people per year, while a large population-based study conducted in France estimated the annual incidence of SCCAD to be 2.6–3 cases per 100,000 people per year [16,17]. The subtle difference between these two incidence rates may be correlated with factors such as climate and the level of medical care provided. The average age at onset is approximately 45 years [18]. Although the frequency of SCCAD increases with age, only 7.1% of patients are older than 60 [4,18,19]. Males are predominant in overall cohorts of SCCAD patients [14,20,21]; However, there is a higher proportion of females among younger patients [18]. The slight male bias in older patients may be attributed to the protective effects of estrogen on cerebral vasculature [22,23].

Phenotypic SCCAD is associated with age, hormones, underlying arterial disease, genetics, inflammation, and environmental stressors [2,18]. Pathological conditions reported in association with patients with SCCAD include: (1) fibromuscular dysplasia [5,24]; (2) pregnancy (postpartum period) [7,25,26]; (3) heritable connective tissue disorders: Marfan syndrome, Loeys–Dietz syndrome, Ehlers–Danlos syndrome, Turner’s syndrome, etc. [6,27,28,29]; (4) infection [6,30,31,32,33]; (5) autoimmune diseases: systemic lupus erythematosus, Takayasu arteritis, Sjögren’s syndrome, autoimmune thyroid disease, myasthenia gravis, etc. [15,34,35]. We selected a subset of case–control studies and cohort studies, presenting the relevant data in Table 1 in chronological order. These studies were chosen based on their extensive data collection, clear presentation, and analysis of clinical symptoms and comorbidities among the study subjects, making them representative of the research field. Despite their valuable insights into rare or unique clinical manifestations, diseases, or treatment outcomes, case reports are subject to selection bias and reporting bias, which may result in potentially inaccurate or incomplete data. Therefore, we did not include further analysis of case reports in our study of clinical manifestations.

Unlike spontaneous coronary artery dissection, exogenous hormones have rarely been reported to be associated with the development of SCCAD [36]. In the context of non-traumatic CCAD, it is noteworthy that there is a dearth of pertinent in vivo study models available. However, some case reports show significant improvement in the clinical profiles of arterial dissection and MRI brain-related lesions in SCCAD patients with the combined autoimmune disease (Takayasu arteritis and SCCAD, cerebral amyloid angiopathy-related inflammation and SCCAD) following the use of high-dose hormones and/or anti-inflammatory drugs [37,38].

This may be due to the differing pathogenesis between these two disease entities. A history of antecedent infection is thought to be significantly associated with SCCAD development, with inflammation potentially playing a critical role [6,30,31,32,33,38,39,40,41,42]. Interestingly, cervicocranial arterial dissection (CAD) is more likely to occur in colder months, regardless of geographic location, suggesting that transient seasonal factors such as infections may play a role in pathophysiology [43,44]. 

Similar to autoimmune diseases, SCCAD results from a complex interaction between genetic and environmental factors [35]. Additionally, the proportion of female SCCAD patients with autoimmune diseases is much higher than that of male patients [14,15]. Sex hormones, particularly estrogen, which is a potent stimulator of autoimmunity, may play a critical role in the gender bias of autoimmune diseases [22]. The female bias in young SCCAD patients is consistent with this observation [18]. 

Spontaneous cervicocranial arterial dissection (SCCAD) can be classified into carotid artery dissection (CAD) and vertebral artery dissection (VAD). Research focusing on European and American populations indicates a higher prevalence of CAD compared to VAD [6,16,45]. Distinct baseline characteristics, locations of onset, and prognoses are observed between these conditions. Notably, male patients are more commonly associated with CAD than VAD [18]. Age also exhibits a correlation, with younger individuals more likely to experience VAD, while older individuals tend towards CAD [18]. Furthermore, CAD predominantly occurs in extracranial arterial segments, whereas VAD is more frequently observed in intracranial arteries [46]. Resolution of stenosis is found to be more common in vertebral dissections compared to carotid dissections [47].

**Table 1:** Clinical characteristics of patients with SCCAD in previous studies.

Authors	Sample Size	Country Origin	Imaging Method	Age	Gender	Anatomical Location	Presenting Symptoms	Multiple Vessels Involved	Concomitant Disease
Biousse, V et al. (1995) [48]	*n* = 80	France	Angiography	range 14 to 67 years	56.3% male	4 (5%) VAD; 80 (100%) extracranial CAD;	neck pain or headache (38.8%); Horner syndrome (18.8%); cerebral ischemic events * (82.5%);	12.50%	1.2% with Ehlers-Danlos syndrome;
Touzé, E et al. (2003) [49]	*n* = 459	France	DSA, MRI, CTA, CUS	44.0 ± 9.7 years	52.9% male	170 (30.7%) sVAD; 384 (51.2%) sCAD;	neck pain and headache and cranial nerve palsy and Horner syndrome (23.3%); SAH (1.1%); cerebral ischemic events * (75.6%);	15.70%	8.7% with fibromuscular dysplasia;
Lee, Vivien H et al. (2006) [20]	*n* = 48	United States	DSA, MRI, CUS	45.8 years	50% male	18 (38%) sVAD; 32 (67%) sCAD;	neck pain (39% sVAD versus 19% sCAD); headache (sVAD 67% versus sCAD 72%); SAH N/A; cranial nerve palsy N/A; Horner syndrome (sVAD 22% versus sCAD 25%); cerebral ischemic events * (sVAD 78% versus sCAD 59%);	13%	6% with indicates connective tissue disorder;
Debette, S et al. (2011) [50]	*n* = 982	Argentina, Belgium, Finland, France, Germany, Italy, Switzerland, Turkey	N/A	sVAD: 41.1 ± 9.9 years; sCAD: 45.7 ± 9.6 years	sVAD: 51.1% male; sCAD: 60.4% male	327 (33.3%) sVAD; 619 (63.0%)sICAD; 36 (3.7%) sVAD + sCAD;	neck pain (66.0% sVAD versus 38.7% sCAD); headache (sVAD 64.5% versus sCAD 67.8%); SAH (sVAD 0.3% versus sCAD 1.0%); cerebral ischemic events * (sVAD 90.2% versus sCAD 73.2%);	15.20%	N/A
Hassan, Ameer E et al. (2011) [51]	*n* = 69	United States	DSA, MRI, CTA	47.8 ± 14.0 years	65.2% male	31 (44.9%) sVAD; 37 (53.6%)sCAD;	in 19 patients with subsequent neurologic deterioration: headache (15.8%); cranial nerve palsy (10.5%); cerebral ischemic events * (78.95%);	13.00%	7.2% with fibromuscular dysplasia;
von Babo, Michelle et al. (2013) [45]	*n* = 970	Switzerland and France	DSA, MRI, CTA, CUS	45.0 ± 10.0 years	59.7% male	302 (31.1%) sVAD; 668 (68.9%)sCAD;	neck pain (65.8% sVAD versus 33.5% sCAD); headache (sVAD 70.4% versus sCAD 71.4%); SAH (sVAD 6.0% versus sCAD 0.6%); cranial nerve palsy (sCAD 9%); Horner syndrome (sCAD 47.2%); cerebral ischemic events * (sVAD 84.4% versus sCAD 70.4%);	bilateral dissection (15.2% sVAD versus 7.6% sICAD)	7.9% with fibromuscular dysplasia; 0.9% with indicates connective tissue disorder;
Li, Hao et al. (2022) [14]	*n* = 215	China	DSA, MRI, CUS	48 (38, 58) years	62.3% male	80 (37.2%) sVAD; 135 (62.8%) sCAD;	cerebral infarction (66%);	12%	12.6% with at least 1 autoimmune disease ^#^;

Note: * cerebral ischemic events mean transient ischemic attack or stroke; ^#^ at least 1 autoimmune disease: participants could have > 1 disease; autoimmune diseases were diagnosed according to the International Classification of Diseases, 9th Revision, and were classified as autoinflammatory diseases and classic autoimmune diseases. Abbreviations: N/A, not applicable; VAD, vertebral artery dissection; CAD, carotid artery dissection; sVAD, spontaneous vertebral artery dissection; sCAD, spontaneous carotid artery dissection; DSA, digital subtraction angiography; MRI, magnetic resonance imaging; CTA, computed tomographic angiography; CUS, carotid ultrasound; SAH, subarachnoid hemorrhage; sICAD, spontaneous internal carotid artery dissection.

## 4. Imaging Characteristics of SCCAD

**Figure 1:**
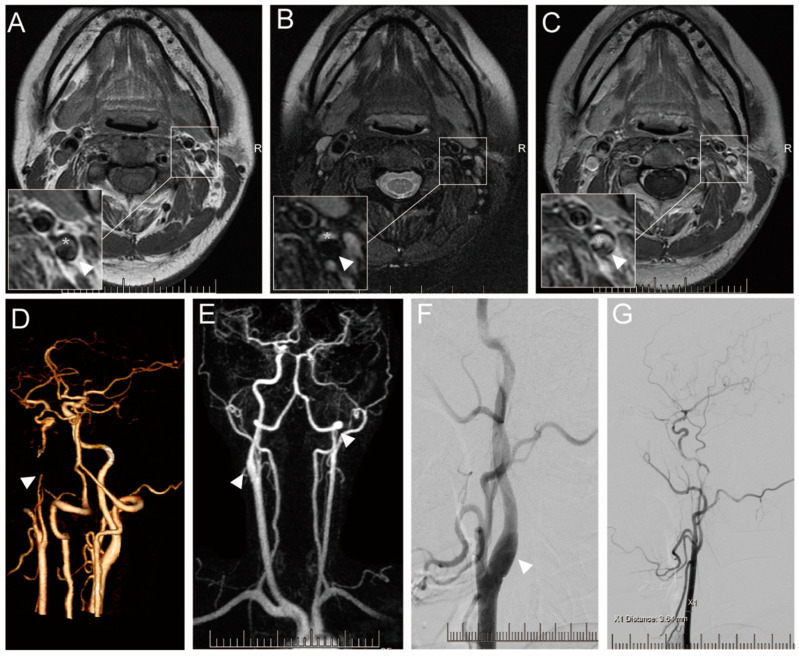
Imaging results of a 36-year-old female with SCCAD. Informed consent was obtained from the patient for the inclusion of their data in this study. Axial T1-weighted (**A**), T2-weighted (**B**), and enhanced T1-weighted (**C**) showing left ICA with hematoma within vascular wall (white asterisk), a true stenotic lumen (white arrowhead). CTA (**D**) shows the occlusion of the left ICA (white arrowhead). HR-MRA (**E**) showing the occlusion of left ICA (white arrowhead) and limited enlarged right ICA with hematoma within the vascular wall. DSA offers the local vascular dissection in the right ICA (**F**) and recanalization of the left ICA (**G**).

The nonspecific symptoms of spontaneous cervicocranial arterial dissection (SCCAD) can closely resemble those of benign migraines, potentially leading to delays in definitive diagnosis or misdiagnosis due to their asymptomatic nature [52,53,54]. Neurological function deterioration may occur even one month after the onset of dissection [9,48,55,56]. Consequently, early and accurate diagnosis is crucial for timely treatment of SCCAD. Various imaging modalities—including computed tomographic angiography (CTA), magnetic resonance imaging (MRI), ultrasound, and digital subtraction angiography (DSA)—can be utilized to identify SCCAD (Figure 1; original illustrations were created by the author to visually represent the concepts discussed in this study). SCCAD imaging features comprise intimal flap, double lumen, tapering stenosis, pseudoaneurysm, and intramural hematoma [57,58,59]. Each neuroimaging modality presents its own advantages and disadvantages based on the clinical scenario.

Digital subtraction angiography (DSA) is the most invasive vascular imaging technique, enabling dynamic characterization of blood flow across the lesion. It is considered the gold standard for diagnosing SCCAD [60]. However, DSA has notable limitations, such as high costs and additional risks, including vascular perforation, stroke, retroperitoneal hemorrhage, and contrast-induced nephropathy [56,58,59].

Compared to DSA, ultrasound serves as a valuable screening tool in clinical settings due to its non-contrast agent usage, radiation absence, and non-invasive nature. Ultrasound can visualize dissection signs, such as an intimal flap or a thickened hypoechoic wall corresponding to mural hematoma, and detect stenosis or occlusion of blood vessels [58]. However, ultrasound requires a high level of skill from the physician and cannot detect dissections above the angle of the mandible [12,61]. Consequently, it is essential to employ additional imaging modalities for further diagnosis [62]. 

Primary advantages of CTA lie in its faster acquisition speed compared to other imaging examinations, making it suitable for imaging clinically unstable patients [59]. In clinical practice, CTA is often used to diagnose arterial dissection (AD) by detecting arterial lumen irregularities [63]. Axial source images and their 3D reconstructions facilitate the identification of intimal tears and accompanying medial or subendothelial hematoma [64]. Given the small diameter of the vertebral artery and its proximity to bony structures, CTA may slightly outperform MRI and ultrasound for VAD [65]. Additionally, CTP can provide information on resultant distal intracranial hemodynamics in acute dissection settings [59]. However, CTA can pose challenges for patients with contrast agent allergies and those at risk of renal impairment. Moreover, CTA is relatively contraindicated for children and pregnant patients [59,65]. 

Despite a longer examination time, MRI is a more suitable choice for patients who cannot tolerate iodinated contrast agents and radiation. In recent years, magnetic resonance angiography/fat saturation images (MRA/FSIs) have been extensively researched and demonstrated a high degree of accuracy in some studies [59]. The advantage primarily lies in assessing intramural hematomas; however, hematomas during the hyperacute phase may still be overlooked [59,66]. Nonetheless, the American Heart Association (AHA), American Stroke Association (ASA), and International Headache Society recommend MRA/FSI as the best initial screening method [61,67]. Additionally, recent studies have employed MRI to identify the age of vessel wall hematomas, which may prove useful for further AD diagnosis and determining its pathogenesis in the future [9,67].

## 5. Clinical Manifestation

The clinical manifestations of SCCAD vary depending on the specific arteries involved, with stenosis being more prevalent than complete occlusion in most patients [6]. In 12–16% of cases, SCCAD involves multiple vessels [14,20,42]. 

CAD typically begins with ipsilateral neck pain or headache and partial Horner’s syndrome (without anhidrosis), followed by retinal or cerebral ischemia [3,37]. Headaches caused by dissection lack specific features, often characterized by sudden-onset, unilateral, constant, and throbbing pain [20,45]. Pain can be isolated or signal an impending stroke [66]. Although SCCAD may mimic a benign headache, some patients remain asymptomatic for extended periods [66]. This observation may be attributed to the periarterial edema discussed previously, wherein inflammation and the resulting edema contribute to irritation and compression of the surrounding tissues, subsequently leading to pain. Neurological symptoms may emerge within one month of arterial dissection onset [9,48,55,56], with the average time from event to symptom appearance being 2 to 3 days [48]. Concurrently, focal neurological symptoms due to retinal or cerebral ischemia may be transient, persistent, or variable [3]. Correspondingly, VAD is more frequently associated with occipito-cervical pain [50]. According to the Cervical Artery Dissection and Ischemic Stroke Patients (CADISP) consortium, patients with VAD are twice as likely to have occipito-cervical pain compared to those with CAD [50,68]. This occurrence may arise from the close proximity of the vertebral artery to the bone structure, as well as its narrower periarterial space. Furthermore, VAD patients often exhibit symptoms of posterior circulation ischemia, including vertigo, dysarthria, visual field deficit, ataxia, and diplopia [3,45]. The role of inflammatory and immune factors in the clinical presentation remains uncertain, but their involvement may potentially exacerbate pain and contribute to the development of dissection tears, ultimately leading to neurological deficits. Patients with SCCAD remain at risk for new or recurrent ischemic events after treatment [3]. Research indicates that female patients with vertebral artery involvement, particularly bilaterally, and those with intracranial artery involvement, exhibit a significantly higher incidence of neurological decline [69]. The clinical characteristics of SCCAD patients are presented in Table 1.The diagnosis and optimal treatment of SCCAD remain highly controversial, potentially due to the complexity of its clinical presentation. Thus, when evaluating patients with headache and neck pain complaints, clinicians should not dismiss the possibility of AD, even in the absence of focal neurological symptoms. Appropriate AD screening is essential in such cases.

A new classification system, the Borgess classification, has been proposed by researchers, which is based on the presence or absence of an intimal tear, as depicted on imaging studies, and its impact on blood flow [70,71]. This classification divides SCCAD into two types, potentially aiding in predicting clinical presentation, prognosis, and guiding clinical management [71]. However, further large-scale prospective studies are necessary to validate its utility.

## 6. Pathophysiology of SCCAD

**Figure 2:**
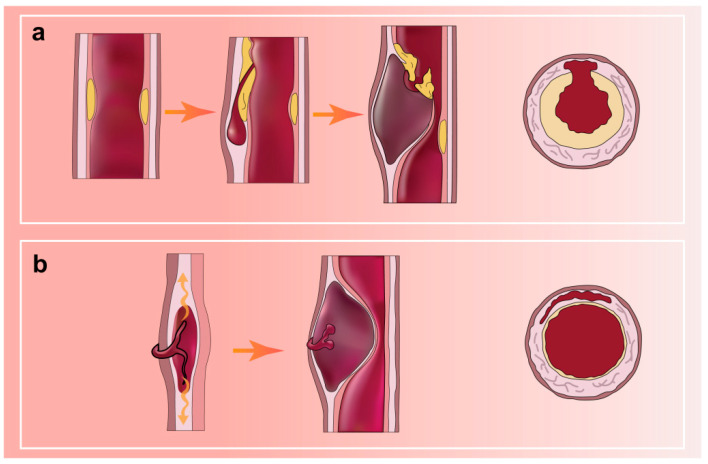
(**a**) AD with a distinct medial tear; (**b**) AD with hemorrhage in the vessel wall but no entry tear.

Arterial dissection, a spontaneous event that can occur in almost all large and medium-sized arteries, is defined as the longitudinal splitting of the arterial wall caused by intramural bleeding [72]. It is characterized by a hematoma within the wall of the internal carotid or vertebral artery. In most arterial dissections, a distinct intimomedial tear is identified (Figure 2a; original illustrations were created by the author to visually represent the concepts discussed in this study). Less commonly, there is hemorrhage within the vessel wall from the vasa vasorum in the dissection, but no visible laceration (Figure 2b; original illustrations were created by the author to visually represent the concepts discussed in this study). Hematomas and thrombi can lead to narrower lumens and occlusions, which may further trigger reduced blood flow and subsequent ischemic strokes [12,73]. The clinical presentation is highly heterogeneous. Although hypoperfusion is a potential cause of ischemia, thromboembolism has been found to cause most ischemic strokes in patients with SCCAD [74]. 

However, most relevant pathophysiology studies have focused on thoracic aortic aneurysms and dissections, with SCCAD-related studies remaining relatively scarce. Given the limited understanding of SCCAD’s pathophysiological mechanisms, the Cervical Artery Dissection and Ischemic Stroke Patients (CADISP) consortium was formed in 2009 to further investigate the disease entity [50,68]. The researchers proposed several hypotheses and sought relevant evidence to support them [75]. Interestingly, similar endothelial dysfunction and lesions have been observed in different models of AD (oxidative stress, shear stress, inflammation, and immunity), which may suggest a common underlying mechanism or links between different pathophysiological processes [75,76]. 

The development of AD requires two pathological conditions: medial degeneration and mechanical wall stress [77]. Similar to AD, the histological hallmark of SCCAD is medial degeneration, considered a predisposing lesion to dissection [13,72,78,79]. Medial degeneration constituents include mucoid matrix accumulation, smooth muscle cell and elastic fiber abnormalities, and medial fibrosis [78]. Internal elastic lamina fragmentation is almost immediately followed by intimal hyperplasia [75]. In patients with recurrent SCCAD, vascular staining showed partial absence of intima, deposition of acidic mucopolysaccharides, and increased elastase activity, which may have accelerated collagen degeneration, diminished vascular elasticity, and ultimately increased vascular fragility [75,80,81]. Corresponding changes can also be observed in the smooth muscle cells of the media in the affected area [82]. Specifically, the muscle cell phenotype transforms from a contractile state to a metabolic state [81,82]. This transformation is accompanied by an expansion of space and the appearance of irregularly oriented collagen fibers between some smooth muscle cells [75,81,82]. These alterations in smooth muscle cell function are believed to potentially result in changes to collagen proteins and elastic fibers [75].

Another possible hypothesis is that SCCAD patients exhibit abnormalities in vascular connective tissue, such as smooth muscle and elastic fibers. SCCAD is characterized by tearing of the intima or rupture of the vasa vasorum, resulting in bleeding within the media. Histologically, the cervicocranial arteries consist of adventitia, media, and intima layers [42]. Among them, the intima and media provide essential structural and mechanical protection to the vascular wall [41]. There is some correlation between the morphological types of VAD and vertebral artery hypoplasia (VAH) [83]. In a related study, skin biopsies were performed on macroscopically normal skin of patients with SCCAD, revealing alterations in the collagen and elastic fiber network in 85% of the cases [84]. Moreover, compared to healthy controls, the results of skin punch biopsies (using a cutting-edge quantitative proteomics approach) in patients with recurrent SCCAD showed significant differences in protein expression associated with the structural integrity of connective tissue or connective tissue disorders [85]. Meanwhile, fibromuscular dysplasia (FMD), characterized by impaired development of smooth muscle fibers within the arterial wall, has been observed as a comorbidity in some patients with arterial dissection [6,45,86]. In fact, different histological patterns of fibrous dysplasia may be more suitable for representing the continuous stages of arteriopathy evolution [75]. The pathological presentation may be progressive.

Meanwhile, the hypothesis that arterial disease may be a phenotypic expression of a general activation of immunity warrants further investigation. Inflammation may play an early role in the pathogenesis of arterial dissection. Chronic infectious aortitis has been found to coexist with mucocutaneous degeneration in patients with aortic dissection [87]. Recent studies also suggest that medial degeneration may result in SCCAD occurrence due to chronic adventitial inflammation caused by the influx of inflammatory cells and further pathological vascular remodeling [42]. One possible explanation is that the initial degenerative process involves a local inflammatory response promoted by granulocytes, particularly neutrophils, which release elastase and collagenase [72,88]. The association of arterial dissection with systemic (especially inflammatory) factors and autoimmune diseases, and the mechanisms involved, have been the focus of numerous studies. The key question is whether this vascular degeneration and chronic remodeling, caused by chronic inflammation, are associated with autoimmune mechanisms. It is plausible that a causal relationship or at least some shared pathophysiological links occur in the same patients. Recent studies have partially confirmed this hypothesis based on gene expression profiles [89]. Medial degeneration within the aortic dissection can impact all of its components. Furthermore, in samples with arterial dissection, a decrease in gene mRNA for elastin and gene expression for actin was observed. Moreover, aortic dissections were associated with an upregulation in the expression of several genes involved in chronic inflammation, including the T-cell costimulatory molecule CD86/B7-2 antigen (43-fold increase), apolipoprotein E (15-fold increase), IL-8 (7-fold increase), GATA-3 (7-fold increase), nuclear factor of activated T cells (6-fold increase), and myeloid cell nuclear differentiation antigen (5-fold increase) [90,91]. 

Tissue samples taken from the macroscopically intact aortic wall of the same patient exhibited highly similar expression profiles [90,91]. Similar to SCCAD, a high degree of heterogeneity, both between diseases and within a single disease, is a major characteristic of autoimmune diseases (AIDs) [92,93]. In patients with AIDs, remodeling of the immune system and chronic systemic inflammation occur [89]. AID can be systemic or affect specific organs or body systems, including the endocrine, gastrointestinal and liver, and neurological systems [89]. The specific clinical presentation often depends on the involved system. However, the shared chronic inflammatory process particularly impacts the cardiovascular system. Multiple factors may contribute to the progressive disruption of the connective components of the vessel wall and weakening of the artery [89]. 

## 7. Etiologies and Risk Factors of SCCAD

Previous studies have suggested that SCCAD may be caused by various factors that contribute to the weakness or stress of the arterial wall [4,28,94]. Hypertension, pregnancy, and recent history of infection have demonstrated a significant correlation [6,7,26,32]. However, many ischemic-stroke-related risk factors, such as hypercholesterolemia and obesity, have been found to have an inverse relationship with CCAD [4].

### 7.1. SCCAD and Traumas

In approximately 40% of SCCAD patients, the onset of symptoms is preceded by mild traumas, while the other half of patients do not report such triggers [6,16]. Most of these events involve minor and insignificant traumas to the head and/or neck [6,12]. Moreover, many cases of SCCAD occur without any history of trauma [12]. Therefore, establishing a causal relationship between minor trauma and SCCAD is often challenging, and other predisposing factors are likely to be involved in the development of the disease. Furthermore, the notion of potential susceptibility is reinforced by numerous other studies. For example, patients with SCCAD were also more likely to have intracranial aneurysms, aortic root dilatation, aortic dissection, and arterial redundancies [8,24,95,96]. In another case–control study, compared to accident victims, patients with spontaneous dissection were more likely to have abnormalities in the adventitia and medial layers of temporal arteries [88]. Interestingly, in elderly patients (>60 years old), SCCAD is frequently painless and without prior mechanical triggering events [4,16,19]. Therefore, it is believed that weak arterial walls, rather than minor traumas, are a potentially important contributing factor in the development of SCCAD.

Based on this hypothesis, case–control studies were conducted by the investigators to examine the potential association between predisposing factors that contribute to the weakening of the vessel wall, such as higher arterial curvature, and the development of SCCAD [97,98]. These studies yielded affirmative results, thereby providing empirical support for the proposed hypothesis. Moreover, these findings indirectly reinforce the notion of a positive relationship between the structural weakening of the cervicocerebral arteries and the morbidity associated with SCCAD.

### 7.2. SCCAD and Connective Tissue Abnormalities

**Figure 3:**
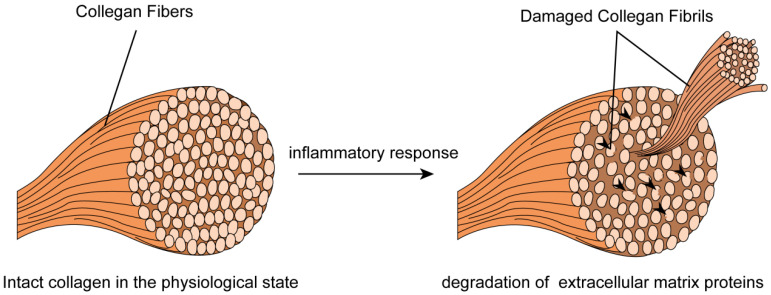
Skin biopsy specimens from SCCAD patients revealed composite collagen fibrils and fragmented elastic fibers.

SCCAD often manifests in healthy individuals lacking known stroke risk factors and frequently arises spontaneously, without related trauma. This suggests that vessel wall weaknesses could stem from underlying arterial lesions. Histopathological and ultrastructural abnormalities, elastic fiber dissection, and medial degeneration have been identified in carotid, aortic, and renal artery specimens from SCCAD patients [99]. Electron microscopic examination of skin biopsy specimens from over half of SCCAD patients revealed composite collagen fibrils and fragmented elastic fibers (Figure 3; original illustrations were created by the author to visually represent the concepts discussed in this study) [10,100]. The observed ultrastructural morphological aberrations in collagen fibers are restricted to elastic fibers, with fragmentation and mini-calcifications without significant alterations to collagen fibril morphology [100]. These aberrations can be classified as “Ehlers-Danlos syndrome (EDS) III-like” or “EDS IV-like” [99]. However, the intricate question remains unanswered regarding whether this morphological alteration signifies a concealed manifestation of a broader connective tissue disorder. Additionally, patients with traumatic SCCAD exhibit a low incidence of clinically discernable connective tissue abnormalities [101]. The hypothesis of an underlying connective tissue disorder is supported by the exclusion of intraindividual variability over time through a second biopsy for some patients with pronounced aberrations [10,100]. The complexity of SCCAD pathogenesis suggests multifactorial influences are at play.

Nonetheless, the prevalence of known connective tissue diseases among SCCAD patients remains low [101,102]. Compared to EDS patients, SCCAD patients exhibited no significant increase in skin extensibility under non-invasive machine measurements [103]. These findings merely suggest an association between carotid artery entrapment and connective tissue abnormalities. The underlying molecular mechanisms could induce structural abnormalities within the extracellular matrix. Clinically detectable connective tissue abnormalities lend indirect support to the “connective hypothesis” of the disease. Further exploration is required to elucidate the association between connective tissue abnormalities in SCCAD patients and systemic autoimmune diseases, also known as CTDs.

### 7.3. Inflammation and SCCAD

**Figure 4:**
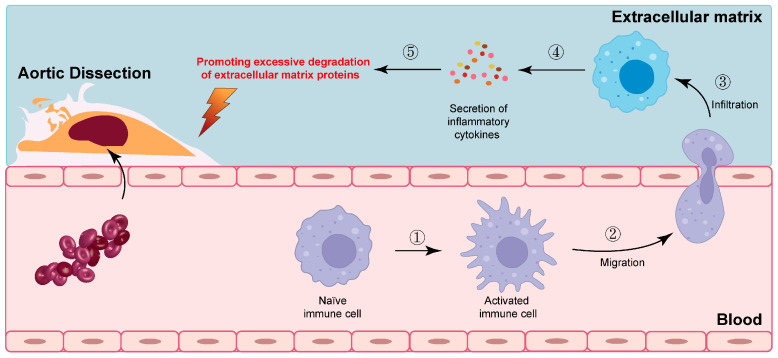
The intricate interplay between pro-inflammatory cytokines, immune cell infiltration, and matrix protein degradation. The numbers in the figure represent specific steps of immune cell activation.

Evidence from biopsies, case reports, and a few case–control studies implies that inflammatory changes may contribute to the cascading events leading to SCCAD [33,38,39,40,41,42]. Upon pathogen infection, local pro-inflammatory cytokines initiate immune-associated inflammatory cell infiltration. Simultaneously, these cytokines induce extensive transient inflammatory arteriopathy by activating protein hydrolysis processes and promoting excessive degradation of extracellular matrix proteins. Consequently, the stability of the arterial wall is compromised, exacerbating the inflammatory response [104,105,106]. The intricate interplay between pro-inflammatory cytokines, immune cell infiltration, and matrix protein degradation underscores the complex nature of the inflammatory response in the context of pathogen-induced vascular inflammation (Figure 4; original illustrations were created by the author to visually represent the concepts discussed in this study).

**Figure 5:**
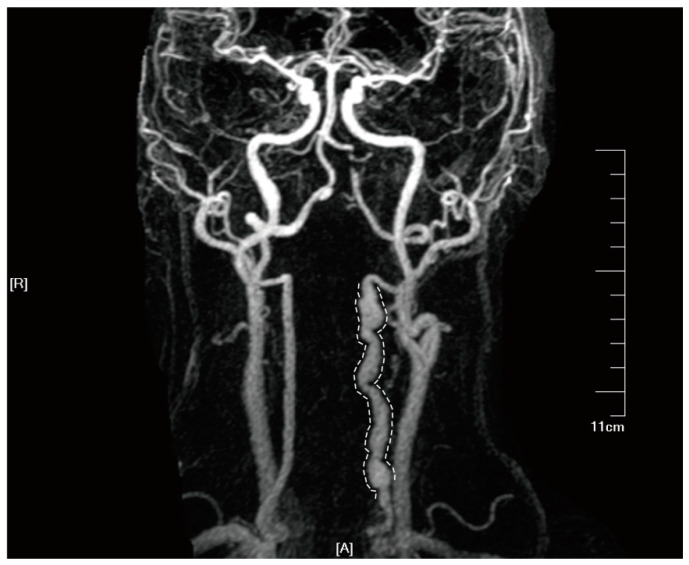
Tortuous and abnormally dilated vertebral artery on HR-MRA imaging in a patient with double vertebral artery dissections. Informed consent was obtained from the patient for the inclusion of their data in this study. In the diagram, letter ‘A’ represents the anterior, and letter ‘R’ denotes the right side.

AD patients often exhibit heterogeneous arterial involvement and multiple degenerative abnormalities (Figure 5; original illustrations were created by the author to visually represent the concepts discussed in this study). Typically, involved arteries display chronic, asymptomatic pathological alterations associated with non-specific features such as arterial stiffness, dilation, and medial degeneration [13,72,83]. These pathological changes predominantly occur chronically, over an extended period before an acute event. The parallels observed in AID and arterial remodeling will be discussed subsequently.

In comparison to traumatic CCAD, SCCAD patients exhibited a higher likelihood of recent infection history, aligning with CADISP study findings, which linked recent infection history to SCCAD development [6]. Infections, such as respiratory tract infections, gastroenteritis, and urinary tract infections, have been identified as potential factors preceding the vascular event, with symptoms typically manifesting within the four weeks prior [107]. Generally, infections tend to be mild and often resolve before hospitalization. In the case of respiratory tract infections, mechanical factors resulting from forceful coughing, sneezing, or vomiting do not appear to account for the observed association with spontaneous SCCAD. Moreover, direct vessel wall damage and cellular infiltration caused by infection seem unlikely. Instead, the indirect effect of inflammation and the role of the immune response may lead to excessive degradation of the extracellular matrix, weakening the vessel wall. In light of this, it gives rise to the potential presence of an underlying connective tissue disorder or fibromuscular dysplasia. 

Prior research has shown that pro-inflammatory cytokines, including interleukins, interferon, tumor necrosis factor superfamily members, colony-stimulating factors, and chemotactic factors, may significantly influence AD by triggering proteolytic processes and facilitating extracellular matrix protein degradation [104,105]. Concurrently, emerging evidence from in vivo pathological investigations in patients and experimental studies utilizing the angiotensin II (Ang II)-induced mouse model of aortic dissection (AD) has illuminated the intricate involvement of an extensive repertoire of cytokines within the cytokine family [104,105]. Virtually all members of this cytokine family have been implicated in the multifaceted development of AD, operating through intricate interactions and signaling pathways. Instead of singular cytokine expression, it is the collective orchestration of these cytokines that contributes to the pathogenesis of AD [105]. Furthermore, the intricate balance between pro-inflammatory and anti-inflammatory effects within the aortic wall emerges as a pivotal determinant in the complex and dynamic progression of AD [104,105].

Pathological and imaging data reveal inflammatory infiltration within arterial walls of intracranial dissections, suggesting local inflammatory alterations as a key factor in SCCAD [37,38]. SCCAD biopsy samples displayed extensive arterial damage and immune-related inflammatory infiltration, contributing to arterial wall stability impairment [106]. Notably, this inflammatory infiltrate is absent in traumatic CCAD and subsides following immunosuppressive therapy (also observed in spontaneous coronary artery dissection) [15,32,38,42,106,108]. The notion of underlying arterial inflammation in SCCAD is further substantiated by the higher association of SCCAD with mural hematoma and periarterial edema compared to traumatic CCAD [32]. Intriguingly, select case reports demonstrated aspirin/steroid treatment leading to remarkable improvements in both clinical condition and magnetic resonance imaging brain lesion presentation [37,38,109]. Nevertheless, discerning whether the inflammatory response is the cause or consequence of histopathological alterations in SCCAD remains a challenge.

Imaging studies have revealed novel insights. Fluorodeoxyglucose (FDG) uptakes were detected along the contralateral, unaffected arteries in SCCAD patients undergoing positron emission tomography (PET) [13,72]. Indications of generalized transient inflammatory arteriopathy were identified in hrMRI and PET-CT for a subset of SCCAD patients who were potentially more susceptible to multiple dissections [13]. Concurrently, inflammation-related genes exhibited differential expression in tissue samples from aortic dissection and intracranial aneurysm [90,91,93,110]. A notable example is the upregulation of MMP9, implicated in elastin degradation in aneurysmal disease due to its enzymatic activity against elastic fibers and other extracellular matrix proteins, and its production by infiltrating macrophages [111]. These findings imply that the observed inflammatory signals along the affected artery are not merely a consequence of reactive inflammation following the dissection event. The absence of a specific infectious agent has prompted the hypothesis that the activation of particular immune-mediated mechanisms, rather than specific infectious factors, may instigate local inflammatory alterations associated with SCCAD.

### 7.4. SCCAD and Autoimmune Diseases

A multitude of case reports suggest a co-occurrence between CCAD and some of the rarer autoimmune diseases [34,112,113,114,115,116,117]. A recent study examined the association between SCCAD and thyroid autoimmunity [15]. Another investigation probed the potential connection between SCCAD and 25 autoimmune diseases [14]. In line with this, autoimmune thyroid disease was found to be the most common concomitant immune disorder observed in this study [14]. This observation can be attributed to the remarkable prevalence of thyroid autoimmunity, which stands as the most prevailing form of autoimmune disease. Both studies yielded supportive data, indicating a significant correlation between autoimmunity and SCCAD.

Autoimmunity is considered a pathogenic mechanism in certain disease entities, such as segmental arterial mediolysis, which is associated with SCCAD occurrence [15,106]. Relative to the general population, patients with AID exhibit an elevated absolute risk of cardiovascular disease, manifesting from early stages of life [118,119]. In AID patients, underlying microangiopathy and macroangiopathy coincide with inflammatory mediator production in perivascular layers, including lipid particle accumulation, autoantibodies, autoantigens, and multiple inflammatory cytokines [118,119,120]. Distinct vascular inflammatory conditions tend to target specific vascular beds, each characterized by a unique, complex pathogenesis [121]. These findings have elucidated vessel-specific risks for inflammatory vasculopathies and enhanced our comprehension of AD susceptibility.

**Figure 6:**
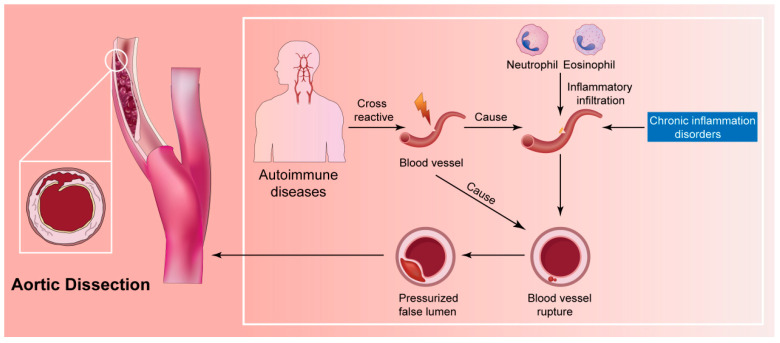
The inflammatory hypothesis for the pathophysiology of SCCAD.

Nonetheless, is the chronic inflammation resulting from autoimmunity directly linked to SCCAD pathogenesis? One conjecture suggests potential cross-reactivity between various tissues, while another proposes that autoimmune antibodies may not directly impact the dissection’s pathogenic process, but instead contribute to the local vasculopathy underlying SCCAD (Figure 6; original illustrations were created by the author to visually represent the concepts discussed in this study).

**Figure 7:**
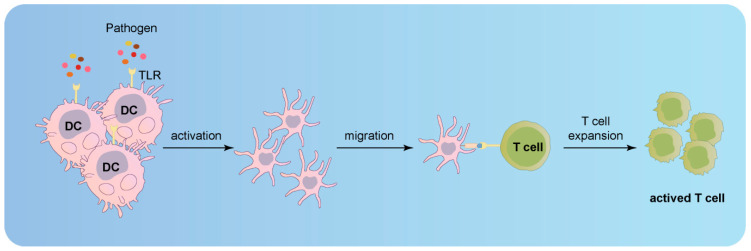
Activation of infiltrating T cells in the vessel wall upon stimulation of Toll-like receptor ligands in DCs.

Electron microscopic alterations were identified in skin biopsies from patients with CCAD and intracranial aneurysms, implying AD may represent a systemic disease manifestation [10,100,122]. However, patients infrequently exhibit multiple ADs in disparate arteries but often present bilateral dissections in carotid, vertebral, renal, or coronary arteries [122,123,124]. Consequently, specific risk factors may pertain to particular arterial segments. Chronic inflammation plays a crucial role in autoimmune diseases, particularly affecting the cardiovascular system [93]. Human arterial walls house dendritic cells (DC) within the wall structure [125]. Experimental investigations involving vessel walls devoid of the intimal or adventitial layer revealed that DCs at the media–adventitia junction serve as the primary pathogen sensors [126]. As immune cells, distinct arterial bed DCs can recognize varying danger signals in the presence of immune stimulation [125]. The aorta and carotid, the largest human arteries, harbor two separate wall-integrated DC networks [125]. Furthermore, each individual vessel within the macrovascular tree exhibits a distinct and intricate TLR profile, which imparts unique immunological profiles specific to each vascular region [126,127]. Upon stimulation of the DCs residing within the vessel wall with Toll-like receptor ligands, the infiltrating T cells within the wall manifest a discerning activation response that is tightly linked to the vasculature itself [127]. Remarkably, this particular vessel under examination exemplifies a TLR profile that substantiates a selectively tailored T-cell response, potentially fostering the development of an intricately orchestrated vessel-specific inflammatory vasculopathy (Figure 7; original illustrations were created by the author to visually represent the concepts discussed in this study) [127].

Genetic and environmental risk factors demonstrate differences between aortic and carotid regions, while other research emphasizes the focal nature of medial degeneration (SCCAD’s histological hallmark) [126]. This also indicates the coexistence of highly affected and less affected areas within the same arterial segment.

Although several case–control studies have demonstrated an association between autoimmunity and SCCAD, the exact mechanisms of both hypotheses remain unverified [14,15]. Definitive mechanistic findings are scarce, with only indirect evidence offering support. In conjunction with the prior discussion, immune-mediated process activation may participate in SCCAD pathogenesis. Autoimmunity could be postulated as a potential biological determinant of SCCAD.

### 7.5. SCCAD and Genetic Disorders

The potential role of genetic alterations in SCCAD pathogenesis remains uncertain. Although relatively rare, familial aggregation is observed in some SCCAD patients [2,28,86]. In patients with a family history of SCCAD, the high incidence of multiple dissection events and long-term (>1 year) recurrent dissections suggest a specific susceptibility to familial SCCAD [128]. This could be coincidental; however, it also raises the hypothesis that SCCAD may be associated with shared genetic factors among affected relatives. Nevertheless, whole-exome sequencing reveals that dissection-related variants are rare in familial SCCAD patients, and samples exhibit high genetic heterogeneity [2,129]. While genetic imbalance may contribute to SCCAD development, it appears to be primarily linked to familial or recurrent cases, as noted in prior studies [130,131,132].

Although most SCCAD cases do not present a recognizable monogenic disorder, evidence exists for an association between SCCAD and certain monogenic connective tissue disorders, particularly vascular Ehlers–Danlos syndrome [10]. The link between SCCAD and hereditary skin connective tissue abnormalities suggests that genetic factors, as part of a multifactorial susceptibility, also play a role in “sporadic” SCCAD [131]. Copy number variants (CNVs) were highly heterogeneous and equally frequent among CCAD patients and controls in the CADISP study [132]. Intriguingly, patients with a family history of CCAD more frequently carried rare CNVs associated with cardiovascular system development [132]. Furthermore, a meta-analysis provided support for a modest correlation between the MTHFR 677TT genotype and SCCAD. Despite limited statistical power due to the small number and size of studies, and the absence of significant heterogeneity among them, the meta-analysis revealed a significant overall association of the MTHFR 677TT genotype with SCCAD [133]. This genotype has been associated with elevated homocysteine levels, which, in turn, could contribute to the development of SCCAD by causing endothelial damage or influencing the elastic properties of the arterial wall [133].

These observations imply a potential genetic predisposition to SCCAD. However, the small size and number of included studies may introduce an unavoidable bias. Overall, most genetic studies of SCCAD (especially non-familial aggregates) have yielded negative results, despite the interesting hypotheses proposed. Nonetheless, they are clearly under-powered. Further genetic investigations of SCCAD necessitate DNA samples from large multi-center series.

## 8. Treatment

Currently, no trials specifically investigate the prognosis of patients with SCCAD combined with AID, resulting in a lack of data on managing these patients compared to those with SCCAD without AID. Consequently, the primary treatment goal for SCCAD remains the prevention of ischemic complications. Although most SCCAD cases recover spontaneously, patients should receive treatment to prevent potential thromboembolism and hemodynamic complications arising from dissection [134]. In the acute phase, patients meeting the criteria should undergo intravenous thrombolysis and/or mechanical thrombectomy [135,136]. In other cases, anticoagulants or antiplatelets should be considered [134,135]. Barring contraindications, lifelong antiplatelet therapy is a common practice. For patients experiencing recurrent ischemia despite optimal drug treatment, surgery should be contemplated [137]. Controlled clinical trials directly comparing the efficacy of endovascular therapy combined with antithrombotic therapy versus antithrombotic therapy alone in patients with SCCAD are currently lacking, leaving limited evidence to support their relative effectiveness. However, it should be noted that there may exist scenarios where endovascular intervention becomes necessary, particularly in cases where patients are not suitable candidates for anticoagulants or dual antiplatelet agents [138]. Furthermore, the emergence of innovative techniques, such as the utilization of the superficial temporal artery for the treatment of common carotid artery dissection, shows potential in providing additional therapeutic benefits for carefully selected patients [139]. Pseudoaneurysms associated with SCCAD generally pose a low rupture risk and thus typically do not necessitate endovascular surgery in most instances [140,141]. Some of the data are presented in Table 2.

Recent findings indicate a direct or indirect association between SCCAD, chronic inflammation, and AID [14]. Although there is limited evidence from population studies due to the scarcity of cases, relevant case reports have demonstrated positive clinical outcomes when patients with SCCAD and AID were treated with immunosuppressive agents, such as high-dose glucocorticoids [37,38]. The aforementioned observations serve as a poignant reminder of the imperative for clinicians to maintain unwavering vigilance when it comes to recognizing the subtle manifestations of SCCAD in individuals afflicted with autoimmune diseases. Notably, symptoms such as facial and neck pain should serve as red flags, prompting comprehensive screenings for associated AID and genetic mutations. This meticulous approach becomes even more crucial for patients presenting with recurrent or bilateral SCCAD or with a family history of AD, warranting thorough evaluation and prompt intervention. By ensuring timely diagnoses and implementing tailored treatment strategies, healthcare professionals can effectively mitigate the potential for adverse prognostic outcomes, thus offering hope and improved quality of life for affected individuals.

## 9. Limitations

This study has several limitations that should be acknowledged. Firstly, the human SCCAD specimens used in this study were collected during the later stages of dissection formation and development, typically through surgery or autopsy. As a result, information regarding the early developmental stages, which could provide insights into disease progression mechanisms, is not readily available. Additionally, distinguishing whether the observed inflammatory responses in human AD specimens are directly associated with dissection development or merely a result of vessel rupture poses a challenge. Nevertheless, the utilization of case–control studies and genomic expression profiling has provided partial evidence supporting the involvement of inflammatory response pathways in SCCAD. Furthermore, it is important to note that this study did not include in vitro experimental studies of AD due to the limited availability of research in this specific area and potential variations in the underlying causes of AD and CCAD.

## 10. Conclusions and Future Directions

In this review, we emphasize the link between SCCAD, chronic inflammation, and AID, and explore their underlying pathophysiological mechanisms. Although direct evidence is limited, previous studies indirectly support their association. Clinicians should remain vigilant for SCCAD symptoms in individuals with autoimmune diseases. Screening for AID and genetic mutations in high-risk patients is important for timely diagnosis and appropriate treatment to prevent adverse prognosis.

Larger clinical cohort studies, encompassing multicenter investigations, are needed to enhance our comprehension of these connections, considering the variations in AID incidence across different latitudes and races. Additionally, further investigations, including the ongoing development and refinement of animal models and human CCAD specimens, are crucial for a better understanding of the relationship.

## Figures and Tables

**Table 2:** Treatments of patients with SCCAD in previous studies.

Authors	Sample Size	Country Origin	Study Design	Patient Characteristics	Interventions	Outcomes
Brandt T. et al. (2001) [141]	N/A	N/A	review article	cervicocerebral artery dissection	partial thromboplastin time-guided anticoagulation by intravenous heparin followed by anticoagulation with warfarin; surgery is not recommended except for persisting severe stenosis of the ICA.	the recurrence rate for SCCAD varies from 4% to 8%; the recanalization rate of SCCAD is 85%
Stella, N. et al. (2010) [139]	*n* = 1	Italy	case report	an 83-year-old man with a post-carotid endarterectomy left CCA dissection was admitted for a TIA involving the left cerebral hemisphere	the endovascular treatment using the superficial temporal artery as the principal access	all vessels involved were patent at 6-month follow-up
Lin, J. et al. (2016) [136]	*n* = 846	N/A	meta-analysis	846 SCCAD patients were identified from 10 studies (174 with thrombolysis; 672 with non-thrombolysis)	thrombolysis	no significant difference in the proportion of patients with a favorable outcome at 3 months’ follow-up between the thrombolysis and non-thrombolysis groups; non-thrombolysis slightly superior in terms of excellent outcome.
Markus, H. S. et al. (2019) [134]	*n* = 250	Britain, Australia	randomized clinical trial	118 sCAD and 132 sVAD, mean age was 49 years.	antiplatelet agents (126) or anticoagulants (124)	the recurrent stroke rate at 1 year was 2.4%; there was no difference in the presence of residual narrowing or occlusion between those receiving antiplatelet agents (*n* = 56 of 92) vs. those receiving anticoagulants (*n* = 53 of 89)
Debette, S. et al. (2021) [135]	N/A	N/A	guidelin	extracranial artery dissection and intracranial artery dissection	intravenous thrombolysis with alteplase; mechanical thrombectomy; anticoagulants; antiplatelets	N/A
Engelter, S.et al. (2021) [137]	N/A	N/A	review article	cervical and intracranial artery dissection	intravenous thrombolysis with alteplase; mechanical thrombectomy; anticoagulants; antiplatelets	intracranial hemorrhage is higher in intracranial artery dissection
Diana, F. et al. (2022) [138]	*n* = 2	Italy	case report	internal carotid artery dissection	flow diverter	flow diversion was successful in both patients
Keser, Z. et al. (2022) [140]	N/A	N/A	review article	intracranial and extracranial SCCAD	long-term antithrombotic therapy	recurrent ischemic events and dissections are rare and typically occur early

Abbreviations: SCCAD, spontaneous cervicocranial arterial dissection; N/A, not applicable; ICA, internal carotid arteries; sVAD, spontaneous vertebral artery dissection; sCAD, spontaneous carotid artery dissection; TIA, transient ischemic attack; CCA, common carotid artery.

## Data Availability

No new data were created or analyzed in this study. Data sharing is not applicable to this article.

## Supplementary Materials

The following supporting information can be downloaded at: https://www.mdpi.com/article/10.3390/jcm12155132/s1.

## References

[1] Engelter S.T., Traenka C., Lyrer P. (2017). Dissection of Cervical and Cerebral Arteries. Curr. Neurol. Neurosci. Rep..

[2] Debette S., Compter A., Labeyrie M.A., Uyttenboogaart M., Metso T.M., Majersik J.J., Goeggel-Simonetti B., Engelter S.T., Pezzini A., Bijlenga P. (2015). Epidemiology, pathophysiology, diagnosis, and management of intracranial artery dissection. Lancet Neurol..

[3] Blum C.A., Yaghi S. (2015). Cervical Artery Dissection: A Review of the Epidemiology, Pathophysiology, Treatment, and Outcome. Arch. Neurosci..

[4] Garg A., Bathla G., Molian V., Limaye K., Hasan D., Leira E.C., Derdeyn C.P., Adams H.P., Shaban A. (2020). Differential Risk Factors and Outcomes of Ischemic Stroke due to Cervical Artery Dissection in Young Adults. Cerebrovasc. Dis..

[5] Bonacina S., Locatelli M., Mazzoleni V., Pezzini D., Padovani A., Pezzini A. (2022). Spontaneous cervical artery dissection and fibromuscular dysplasia: Epidemiologic and biologic evidence of a mutual relationship. Trends Cardiovasc. Med..

[6] Robertson J.J., Koyfman A. (2016). Cervical Artery Dissections: A Review. J. Emerg. Med..

[7] Salehi Omran S., Parikh N.S., Poisson S., Armstrong J., Merkler A.E., Prabhu M., Navi B.B., Riley L.E., Fink M.E., Kamel H. (2020). Association between Pregnancy and Cervical Artery Dissection. Ann. Neurol..

[8] Venturini G., Vuolo L., Pracucci G., Picchioni A., Failli Y., Benvenuti F., Sarti C. (2022). Association between carotid artery dissection and vascular tortuosity: A case-control study. Neuroradiology.

[9] Debette S. (2014). Pathophysiology and risk factors of cervical artery dissection: What have we learnt from large hospital-based cohorts?. Curr. Opin. Neurol..

[10] Giossi A., Ritelli M., Costa P., Morotti A., Poli L., Del Zotto E., Volonghi I., Chiarelli N., Gamba M., Bovi P. (2014). Connective tissue anomalies in patients with spontaneous cervical artery dissection. Neurology.

[11] Adham S., Billon C., Legrand A., Domigo V., Denarie N., Charpentier E., Jeunemaitre X., Frank M. (2021). Spontaneous Cervical Artery Dissection in Vascular Ehlers-Danlos Syndrome: A Cohort Study. Stroke.

[12] Debette S., Leys D. (2009). Cervical-artery dissections: Predisposing factors, diagnosis, and outcome. Lancet Neurol..

[13] Pfefferkorn T., Saam T., Rominger A., Habs M., Gerdes L.A., Schmidt C., Cyran C., Straube A., Linn J., Nikolaou K. (2011). Vessel wall inflammation in spontaneous cervical artery dissection: A prospective, observational positron emission tomography, computed tomography, and magnetic resonance imaging study. Stroke.

[14] Li H., Song P., Yang W., Yang L., Diao S., Huang S., Wang Y., Xu X., Yang Y. (2021). Association between Autoimmune Diseases and Spontaneous Cervicocranial Arterial Dissection. Front. Immunol..

[15] Pezzini A., Del Zotto E., Mazziotti G., Ruggeri G., Franco F., Giossi A., Giustina A., Padovani A. (2006). Thyroid autoimmunity and spontaneous cervical artery dissection. Stroke.

[16] Bejot Y., Daubail B., Debette S., Durier J., Giroud M. (2014). Incidence and outcome of cerebrovascular events related to cervical artery dissection: The Dijon Stroke Registry. Int. J. Stroke.

[17] Ranjbar M., Badihian N., Yazdi M., Milani S., Taheri M., Khorvash F., Saadatnia M. (2022). Incidence, characteristics and prognosis of cervical artery dissection-induced ischemic stroke in central Iran. BMC Neurol..

[18] Metso T.M., Debette S., Grond-Ginsbach C., Engelter S.T., Leys D., Brandt T., Pezzini A., Bersano A., Kloss M., Thijs V. (2012). Age-dependent differences in cervical artery dissection. J. Neurol..

[19] Traenka C., Dougoud D., Simonetti B.G., Metso T.M., Debette S., Pezzini A., Kloss M., Grond-Ginsbach C., Majersik J.J., Worrall B.B. (2017). Cervical artery dissection in patients ≥60 years: Often painless, few mechanical triggers. Neurology.

[20] Lee V.H., Brown R.D., Mandrekar J.N., Mokri B. (2006). Incidence and outcome of cervical artery dissection: A population-based study. Neurology.

[21] Metso A.J., Metso T.M., Debette S., Dallongeville J., Lyrer P.A., Pezzini A., Lichy C., Kloss M., Brandt T., Touze E. (2012). Gender and cervical artery dissection. Eur. J. Neurol..

[22] Sanz I., Lund F. (2019). Complexity and heterogeneity-the defining features of autoimmune disease. Curr. Opin. Immunol..

[23] Boese A.C., Kim S.C., Yin K.J., Lee J.P., Hamblin M.H. (2017). Sex differences in vascular physiology and pathophysiology: Estrogen and androgen signaling in health and disease. Am. J. Physiol. Heart Circ. Physiol..

[24] Southerland A.M., Meschia J.F., Worrall B.B. (2013). Shared associations of nonatherosclerotic, large-vessel, cerebrovascular arteriopathies: Considering intracranial aneurysms, cervical artery dissection, moyamoya disease and fibromuscular dysplasia. Curr. Opin. Neurol..

[25] Urrutia F., Mazzon E., Brunser A., Diaz V., Calderon J.F., Stecher X., Bernstein T., Zuniga P., Schilling A., Munoz Venturelli P. (2022). Cervical Artery Dissection in Postpartum Women after Cesarean and Vaginal Delivery. J. Stroke Cerebrovasc. Dis..

[26] Arnold M., Camus-Jacqmin M., Stapf C., Ducros A., Viswanathan A., Berthet K., Bousser M.G. (2008). Postpartum cervicocephalic artery dissection. Stroke.

[27] Kalashnikova L.A., Danilova M.S., Gubanova M.V., Dreval M.V., Dobrynina L.A., Chechetkin A.O. (2021). Internal carotid artery dissection in patients with Turner’s syndrome. Zhurnal Nevrol. I Psikhiatrii Im. S.S. Korsakova.

[28] Debette S., Goeggel Simonetti B., Schilling S., Martin J.J., Kloss M., Sarikaya H., Hausser I., Engelter S., Metso T.M., Pezzini A. (2014). Familial occurrence and heritable connective tissue disorders in cervical artery dissection. Neurology.

[29] Debette S., Germain D.P. (2014). Neurologic manifestations of inherited disorders of connective tissue. Handb. Clin. Neurol..

[30] Eberhardt R.T., Dhadly M. (2007). Giant cell arteritis: Diagnosis, management, and cardiovascular implications. Cardiol. Rev..

[31] Iwasa M., Mima Y., Ito A., Abe Y., Ueda N., Otsubo R. (2018). A case of bilateral cervical internal carotid artery dissection following herpes zoster of the trigeminal nerve. Rinsho Shinkeigaku.

[32] Naggara O., Touze E., Marsico R., Leclerc X., Nguyen T., Mas J.L., Pruvo J.P., Meder J.F., Oppenheim C. (2009). High-resolution MR imaging of periarterial edema associated with biological inflammation in spontaneous carotid dissection. Eur. Radiol..

[33] Purdy K., Long R., Jickling G. (2022). Case Report: COVID-19 Infection and Cervical Artery Dissection. Am. J. Trop. Med. Hyg..

[34] Caso V., Paciaroni M., Parnetti L., Cardaioli G., Biscarini L., Acciarini A.E., Rubino S., Gallai V. (2002). Stroke related to carotid artery dissection in a young patient with Takayasu arteritis, systemic lupus erythematosus and antiphospholipid antibody syndrome. Cerebrovasc. Dis..

[35] Ortona E., Pierdominici M., Maselli A., Veroni C., Aloisi F., Shoenfeld Y. (2016). Sex-based differences in autoimmune diseases. Ann. Ist. Super. Sanita.

[36] Kang G. (2020). Spontaneous Coronary Artery Dissection and Exogenous Hormone. Ann. Thorac. Surg..

[37] Herath H., Pahalagamage S.P., Withana D., Senanayake S. (2017). Complete ophthalmoplegia, complete ptosis and dilated pupil due to internal carotid artery dissection: As the first manifestation of Takayasu arteritis. BMC Cardiovasc. Disord..

[38] Saliou V., Ben Salem D., Ognard J., Guellec D., Marcorelles P., Rouhart F., Zagnoli F., Timsit S. (2018). A Collet-Sicard syndrome due to internal carotid artery dissection associated with cerebral amyloid angiopathy-related inflammation. SAGE Open Med. Case Rep..

[39] Hunter M.D., Moon Y.P., Miller E.C., Kulick E.R., Boehme A.K., Elkind M.S. (2021). Influenza-Like Illness is Associated with Increased Short-Term Risk of Cervical Artery Dissection. J. Stroke Cerebrovasc. Dis..

[40] Collamer A.N., Battafarano D. (2013). A pain in the neck: Carotid artery dissection presenting as vasculitis. Mil. Med..

[41] Bunton T.E., Biery N.J., Myers L., Gayraud B., Ramirez F., Dietz H.C. (2001). Phenotypic alteration of vascular smooth muscle cells precedes elastolysis in a mouse model of Marfan syndrome. Circ. Res..

[42] Compter A., Schilling S., Vaineau C.J., Goeggel-Simonetti B., Metso T.M., Southerland A., Pezzini A., Kloss M., Touze E., Worrall B.B. (2018). Determinants and outcome of multiple and early recurrent cervical artery dissections. Neurology.

[43] Thomas L.C., Hall L.A., Attia J.R., Holliday E.G., Markus H.S., Levi C.R. (2017). Seasonal Variation in Spontaneous Cervical Artery Dissection: Comparing between UK and Australian Sites. J. Stroke Cerebrovasc. Dis..

[44] Schievink W.I., Wijdicks E.F., Kuiper J.D. (1998). Seasonal pattern of spontaneous cervical artery dissection. J. Neurosurg..

[45] von Babo M., De Marchis G.M., Sarikaya H., Stapf C., Buffon F., Fischer U., Heldner M.R., Gralla J., Jung S., Simonetti B.G. (2013). Differences and similarities between spontaneous dissections of the internal carotid artery and the vertebral artery. Stroke.

[46] Wu Y., Chen H., Xing S., Tan S., Chen X., Tan Y., Zeng J., Zhang J. (2020). Predisposing factors and radiological features in patients with internal carotid artery dissection or vertebral artery dissection. BMC Neurol..

[47] Redekop G.J. (2008). Extracranial carotid and vertebral artery dissection: A review. Can. J. Neurol. Sci..

[48] Biousse V., D’Anglejan-Chatillon J., Touboul P.J., Amarenco P., Bousser M.G. (1995). Time course of symptoms in extracranial carotid artery dissections. A series of 80 patients. Stroke.

[49] Touzé E., Gauvrit J.Y., Moulin T., Meder J.F., Bracard S., Mas J.L. (2003). Risk of stroke and recurrent dissection after a cervical artery dissection: A multicenter study. Neurology.

[50] Debette S., Grond-Ginsbach C., Bodenant M., Kloss M., Engelter S., Metso T., Pezzini A., Brandt T., Caso V., Touze E. (2011). Differential features of carotid and vertebral artery dissections: The CADISP study. Neurology.

[51] Hassan A.E., Jadhav V., Zacharatos H., Chaudhry S.A., Rodriguez G.J., Mohammad Y.M., Suri M.F., Tariq N., Vazquez G., Tummala R.P. (2013). Determinants of neurologic deterioration and stroke-free survival after spontaneous cervicocranial dissections: A multicenter study. J. Stroke Cerebrovasc. Dis..

[52] Sharif M., Trinick T., Khan K.H. (2010). Identification of internal carotid artery dissection in patients with migraine—Case report and literature review. J. Pak. Med. Assoc..

[53] Tsivgoulis G., Mantatzis M., Vadikolias K., Heliopoulos I., Charalampopoulos K., Mitsoglou A., Georgiadis G.S., Giannopoulos S., Piperidou C. (2013). Internal carotid artery dissection presenting as new-onset cluster headache. Neurol. Sci..

[54] Maruyama H., Nagoya H., Kato Y., Deguchi I., Fukuoka T., Ohe Y., Horiuchi Y., Dembo T., Uchino A., Tanahashi N. (2012). Spontaneous cervicocephalic arterial dissection with headache and neck pain as the only symptom. J. Headache Pain..

[55] Grond-Ginsbach C., Metso T.M., Metso A.J., Pezzini A., Tatlisumak T., Hakimi M., Grau A.J., Kloss M., Lichy C. (2013). Cervical artery dissection goes frequently undiagnosed. Med. Hypotheses.

[56] Ben Hassen W., Machet A., Edjlali-Goujon M., Legrand L., Ladoux A., Mellerio C., Bodiguel E., Gobin-Metteil M.P., Trystram D., Rodriguez-Regent C. (2014). Imaging of cervical artery dissection. Diagn. Interv. Imaging.

[57] Teasdale E., Zampakis P., Santosh C., Razvi S. (2011). Multidetector computed tomography angiography: Application in vertebral artery dissection. Ann. Indian Acad. Neurol..

[58] Medel R., Starke R.M., Valle-Giler E.P., Martin-Schild S., El Khoury R., Dumont A.S. (2014). Diagnosis and treatment of arterial dissections. Curr. Neurol. Neurosci. Rep..

[59] Hakimi R., Sivakumar S. (2019). Imaging of Carotid Dissection. Curr. Pain Headache Rep..

[60] Hanning U., Sporns P.B., Schmiedel M., Ringelstein E.B., Heindel W., Wiendl H., Niederstadt T., Dittrich R. (2017). CT versus MR Techniques in the Detection of Cervical Artery Dissection. J. Neuroimaging.

[61] Brott T.G., Halperin J.L., Abbara S., Bacharach J.M., Barr J.D., Bush R.L., Cates C.U., Creager M.A., Fowler S.B., Friday G. (2011). 2011 ASA/ACCF/AHA/AANN/AANS/ACR/ASNR/CNS/SAIP/SCAI/SIR/SNIS/SVM/SVS guideline on the management of patients with extracranial carotid and vertebral artery disease: Executive summary. A report of the American College of Cardiology Foundation/American Heart Association Task Force on Practice Guidelines, and the American Stroke Association, American Association of Neuroscience Nurses, American Association of Neurological Surgeons, American College of Radiology, American Society of Neuroradiology, Congress of Neurological Surgeons, Society of Atherosclerosis Imaging and Prevention, Society for Cardiovascular Angiography and Interventions, Society of Interventional Radiology, Society of NeuroInterventional Surgery, Society for Vascular Medicine, and Society for Vascular Surgery. Circulation.

[62] Sporns P.B., Niederstadt T., Heindel W., Raschke M.J., Hartensuer R., Dittrich R., Hanning U. (2019). Imaging of Spontaneous and Traumatic Cervical Artery Dissection: Comparison of Typical CT Angiographic Features. Clin. Neuroradiol..

[63] Mozayan M., Sexton C. (2012). Imaging of carotid artery dissection. J. Community Hosp. Intern. Med. Perspect..

[64] Shakir H.J., Davies J.M., Shallwani H., Siddiqui A.H., Levy E.I. (2016). Carotid and Vertebral Dissection Imaging. Curr. Pain Headache Rep..

[65] Ebrahimzadeh S.A., Manzoor K., Edlow J.A., Selim M., Chang Y.M., Bhadelia R.A., Mehta P. (2022). Diagnostic yield of CT angiography performed for suspected cervical artery dissection in the emergency department. Emerg. Radiol..

[66] Headache Classification Committee of the International Headache Society (2013). The International Classification of Headache Disorders, 3rd edition (beta version). Cephalalgia.

[67] Habs M., Pfefferkorn T., Cyran C.C., Grimm J., Rominger A., Hacker M., Opherk C., Reiser M.F., Nikolaou K., Saam T. (2011). Age determination of vessel wall hematoma in spontaneous cervical artery dissection: A multi-sequence 3T cardiovascular magnetic resonance study. J. Cardiovasc. Magn. Reson..

[68] Debette S., Metso T.M., Pezzini A., Engelter S.T., Leys D., Lyrer P., Metso A.J., Brandt T., Kloss M., Lichy C. (2009). CADISP-genetics: An International project searching for genetic risk factors of cervical artery dissections. Int. J. Stroke.

[69] Mazzon E., Rocha D., Brunser A.M., De la Barra C., Stecher X., Bernstein T., Zuniga P., Diaz V., Martinez G., Munoz Venturelli P. (2020). Cervical Artery Dissections with and without stroke, risk factors and prognosis: A Chilean prospective cohort. J. Stroke Cerebrovasc. Dis..

[70] Investigators C.t., Markus H.S., Hayter E., Levi C., Feldman A., Venables G., Norris J. (2015). Antiplatelet treatment compared with anticoagulation treatment for cervical artery dissection (CADISS): A randomised trial. Lancet Neurol..

[71] Perry B.C., Al-Ali F. (2013). Spontaneous cervical artery dissection: The borgess classification. Front. Neurol..

[72] Allaire E., Schneider F., Saucy F., Dai J., Cochennec F., Michineau S., Zidi M., Becquemin J.P., Kirsch M., Gervais M. (2009). New insight in aetiopathogenesis of aortic diseases. Eur. J. Vasc. Endovasc. Surg..

[73] Kim Y.K., Schulman S. (2009). Cervical artery dissection: Pathology, epidemiology and management. Thromb. Res..

[74] Morel A., Naggara O., Touze E., Raymond J., Mas J.L., Meder J.F., Oppenheim C. (2012). Mechanism of ischemic infarct in spontaneous cervical artery dissection. Stroke.

[75] Lelong D.C., Logak M. (2004). Pathogenesis of spontaneous cervico-cerebral artery dissection. A hypothesis and a review of the literature. Med. Hypotheses.

[76] Bax M., Romanov V., Junday K., Giannoulatou E., Martinac B., Kovacic J.C., Liu R., Iismaa S.E., Graham R.M. (2022). Arterial dissections: Common features and new perspectives. Front. Cardiovasc. Med..

[77] Akutsu K. (2019). Etiology of aortic dissection. Gen. Thorac. Cardiovasc. Surg..

[78] Halushka M.K., Angelini A., Bartoloni G., Basso C., Batoroeva L., Bruneval P., Buja L.M., Butany J., d’Amati G., Fallon J.T. (2016). Consensus statement on surgical pathology of the aorta from the Society for Cardiovascular Pathology and the Association For European Cardiovascular Pathology: II. Noninflammatory degenerative diseases-nomenclature and diagnostic criteria. Cardiovasc. Pathol..

[79] Ban E., Cavinato C., Humphrey J.D. (2022). Critical Pressure of Intramural Delamination in Aortic Dissection. Ann. Biomed. Eng..

[80] Ohtoh T., Ono Y., Iwasaki Y., Sakurai Y., Nishino A., Arai H., Suzuki H., Namba Y. (2003). Non-traumatic recurrent dissection and its spontaneous repair in the circle of Willis: Report of two autopsy cases. Neuropathology.

[81] Thal D.R., Schober R., Schlote W. (1997). Carotid artery dissection in a young adult: Cystic medial necrosis associated with an increased elastase content. Clin. Neuropathol..

[82] Nakashima Y., Sueishi K. (1992). Alteration of elastic architecture in the lathyritic rat aorta implies the pathogenesis of aortic dissecting aneurysm. Am. J. Pathol..

[83] Park M., Shin N.Y., Yoo J., Heo J.H., Choi J.H., Cho D.Y., Lee S.K. (2019). Association between morphologic subtypes of vertebral artery dissection and vertebral artery hypoplastic appearance. Eur. J. Radiol..

[84] Ulbricht D., Diederich N.J., Hermanns-Le T., Metz R.J., Macian F., Pierard G.E. (2004). Cervical artery dissection: An atypical presentation with Ehlers-Danlos-like collagen pathology?. Neurology.

[85] Mayer-Suess L., Pechlaner R., Barallobre-Barreiro J., Boehme C., Toell T., Lynch M., Yin X., Willeit J., Gizewski E.R., Perco P. (2020). Extracellular matrix protein signature of recurrent spontaneous cervical artery dissection. Neurology.

[86] Atalay Y.B., Piran P., Chatterjee A., Murthy S., Navi B.B., Liberman A.L., Dardick J., Zhang C., Kamel H., Merkler A.E. (2021). Prevalence of Cervical Artery Dissection Among Hospitalized Patients With Stroke by Age in a Nationally Representative Sample From the United States. Neurology.

[87] Wiskott K., Genet P., Lobrinus J.A., Fracasso T., Lardi C. (2019). Intimomedial mucoid arterial degeneration, a rare arterial disorder of forensic significance. Forensic Sci. Med. Pathol..

[88] Volker W., Dittrich R., Grewe S., Nassenstein I., Csiba L., Herczeg L., Borsay B.A., Robenek H., Kuhlenbaumer G., Ringelstein E.B. (2011). The outer arterial wall layers are primarily affected in spontaneous cervical artery dissection. Neurology.

[89] Grond-Ginsbach C., Debette S. (2009). The association of connective tissue disorders with cervical artery dissections. Curr. Mol. Med..

[90] Absi T.S., Sundt T.M., Tung W.S., Moon M., Lee J.K., Damiano R.R., Thompson R.W. (2003). Altered patterns of gene expression distinguishing ascending aortic aneurysms from abdominal aortic aneurysms: Complementary DNA expression profiling in the molecular characterization of aortic disease. J. Thorac. Cardiovasc. Surg..

[91] Muller B.T., Modlich O., Prisack H.B., Bojar H., Schipke J.D., Goecke T., Feindt P., Petzold T., Gams E., Muller W. (2002). Gene expression profiles in the acutely dissected human aorta. Eur. J. Vasc. Endovasc. Surg..

[92] Larsson S.C., King A., Madigan J., Levi C., Norris J.W., Markus H.S. (2017). Prognosis of carotid dissecting aneurysms: Results from CADISS and a systematic review. Neurology.

[93] Wang L., Wang F.S., Gershwin M.E. (2015). Human autoimmune diseases: A comprehensive update. J. Intern. Med..

[94] Engelter S.T., Grond-Ginsbach C., Metso T.M., Metso A.J., Kloss M., Debette S., Leys D., Grau A., Dallongeville J., Bodenant M. (2013). Cervical artery dissection: Trauma and other potential mechanical trigger events. Neurology.

[95] Tzourio C., Cohen A., Lamisse N., Biousse V., Bousser M.G. (1997). Aortic root dilatation in patients with spontaneous cervical artery dissection. Circulation.

[96] Witsch J., Mir S.A., Parikh N.S., Murthy S.B., Kamel H., Navi B.B., Segal A.Z., Fink M.E., Rutrick S.B., Safford M.M. (2021). Association Between Cervical Artery Dissection and Aortic Dissection. Circulation.

[97] Giossi A., Mardighian D., Caria F., Poli L., De Giuli V., Costa P., Morotti A., Gamba M., Gilberti N., Ritelli M. (2017). Arterial tortuosity in patients with spontaneous cervical artery dissection. Neuroradiology.

[98] Kim B.J., Yang E., Kim N.Y., Kim M.J., Kang D.W., Kwon S.U., Kim J.S. (2016). Vascular Tortuosity May Be Associated With Cervical Artery Dissection. Stroke.

[99] Brandt T., Morcher M., Hausser I. (2005). Association of cervical artery dissection with connective tissue abnormalities in skin and arteries. Front. Neurol. Neurosci..

[100] Brandt T., Orberk E., Weber R., Werner I., Busse O., Muller B.T., Wigger F., Grau A., Grond-Ginsbach C., Hausser I. (2001). Pathogenesis of cervical artery dissections: Association with connective tissue abnormalities. Neurology.

[101] Dittrich R., Heidbreder A., Rohsbach D., Schmalhorst J., Nassenstein I., Maintz D., Ringelstein E.B., Nabavi D.G., Kuhlenbaumer G. (2007). Connective tissue and vascular phenotype in patients with cervical artery dissection. Neurology.

[102] Yerramilli S.K., Kokula P., Gupta S.K., Radotra B.D., Aggarwal A., Aggarwal D., Chatterjee D. (2020). Connective Tissue Abnormalities in Patients with Ruptured Intracranial Aneurysms and No Known Systemic Connective Tissue Disorder. World Neurosurg..

[103] Heidbreder A.E., Ringelstein E.B., Dittrich R., Nabavi D., Metze D., Kuhlenbaumer G. (2008). Assessment of skin extensibility and joint hypermobility in patients with spontaneous cervical artery dissection and Ehlers-Danlos syndrome. J. Clin. Neurosci..

[104] Zeng T., Shi L., Ji Q., Shi Y., Huang Y., Liu Y., Gan J., Yuan J., Lu Z., Xue Y. (2018). Cytokines in aortic dissection. Clin. Chim. Acta.

[105] Choke E., Cockerill G.W., Laing K., Dawson J., Wilson W.R., Loftus I.M., Thompson M.M. (2009). Whole genome-expression profiling reveals a role for immune and inflammatory response in abdominal aortic aneurysm rupture. Eur. J. Vasc. Endovasc. Surg..

[106] Volker W., Besselmann M., Dittrich R., Nabavi D., Konrad C., Dziewas R., Evers S., Grewe S., Kramer S.C., Bachmann R. (2005). Generalized arteriopathy in patients with cervical artery dissection. Neurology.

[107] Guillon B., Berthet K., Benslamia L., Bertrand M., Bousser M.G., Tzourio C. (2003). Infection and the risk of spontaneous cervical artery dissection: A case-control study. Stroke.

[108] Koller P.T., Cliffe C.M., Ridley D.J. (1998). Immunosuppressive therapy for peripartum-type spontaneous coronary artery dissection: Case report and review. Clin. Cardiol..

[109] Forster K., Poppert H., Conrad B., Sander D. (2006). Elevated inflammatory laboratory parameters in spontaneous cervical artery dissection as compared to traumatic dissection: A retrospective case-control study. J. Neurol..

[110] Kimura N., Futamura K., Arakawa M., Okada N., Emrich F., Okamura H., Sato T., Shudo Y., Koyano T.K., Yamaguchi A. (2017). Gene expression profiling of acute type A aortic dissection combined with in vitro assessment. Eur. J. Cardiothorac. Surg..

[111] Takai S., Jin D. (2022). Pathophysiological Role of Chymase-Activated Matrix Metalloproteinase-9. Biomedicines.

[112] Kong F., Xia M., Zhao Y., Hua Y., Su L., Li X. (2021). Carotid Artery Dissection: A Rare Presenting Manifestation of Takayasu Arteritis. J. Clin. Rheumatol..

[113] Aubart M., Gobert D., Aubart-Cohen F., Detaint D., Hanna N., d’Indya H., Lequintrec J.S., Renard P., Vigneron A.M., Dieude P. (2014). Early-onset osteoarthritis, Charcot-Marie-Tooth like neuropathy, autoimmune features, multiple arterial aneurysms and dissections: An unrecognized and life threatening condition. PLoS ONE.

[114] Iseki T., Yamashita Y., Ueno Y., Hira K., Miyamoto N., Yamashiro K., Tsunemi T., Teranishi K., Yatomi K., Nakajima S. (2021). Cerebral artery dissection secondary to antiphospholipid syndrome: A report of two cases and a literature review. Lupus.

[115] Oncel C., Kiroglu Y., Erdogan C., Can I., Bir L.S. (2011). Sjogren’s syndrome and vertebral artery dissection. J. Emerg. Med..

[116] Ozen S., Guzel S. (2022). Multiple dissecting intracranial and extracranial aneurysms in rheumatoid arthritis: A rare case. Int. J. Neurosci..

[117] Durand-Dubief F., Marignier R., Berthezene Y., Cottin J., Nighoghossian N., Vukusic S. (2020). Spontaneous multiple cervical artery dissections after alemtuzumab. Mult. Scler..

[118] Castaneda S., Vicente-Rabaneda E.F., Garcia-Castaneda N., Prieto-Pena D., Dessein P.H., Gonzalez-Gay M.A. (2020). Unmet needs in the management of cardiovascular risk in inflammatory joint diseases. Expert Rev. Clin. Immunol..

[119] Lopez-Mejias R., Castaneda S., Gonzalez-Juanatey C., Corrales A., Ferraz-Amaro I., Genre F., Remuzgo-Martinez S., Rodriguez-Rodriguez L., Blanco R., Llorca J. (2016). Cardiovascular risk assessment in patients with rheumatoid arthritis: The relevance of clinical, genetic and serological markers. Autoimmun. Rev..

[120] Gonzalez-Gay M.A., Gonzalez-Juanatey C. (2012). Inflammation, endothelial function and atherosclerosis in rheumatoid arthritis. Arthritis Res. Ther..

[121] Weyand C.M., Goronzy J.J. (2009). Pathogenesis of medium- and large-vessel vasculitis. Z. Rheumatol..

[122] Censori B., Agostinis C., Partziguian T., Guagliumi G., Bonaldi G., Poloni M. (2004). Spontaneous dissection of carotid and coronary arteries. Neurology.

[123] Marshman L.A., Ball L., Jadun C.K. (2007). Spontaneous bilateral carotid and vertebral artery dissections associated with multiple disparate intracranial aneurysms, subarachnoid hemorrhage and spontaneous resolution. Case report and literature review. Clin. Neurol. Neurosurg..

[124] Wakino S., Tawarahara K., Tsuchiya N., Kurosawa Y., Sugihara T., Ando K. (2005). Spontaneous multiple arterial dissections presenting with renal infarction and subarachnoid hemorrhage in a patient under treatment for infertility. Circ. J..

[125] Bobryshev Iu V., Karagodin V.P., Orekhov A.N. (2012). Dendritic cells and their role in immune reactions of atherosclerosis. Tsitologiia.

[126] Grond-Ginsbach C., Pjontek R., Aksay S.S., Hyhlik-Durr A., Bockler D., Gross-Weissmann M.L. (2010). Spontaneous arterial dissection: Phenotype and molecular pathogenesis. Cell. Mol. Life Sci..

[127] Pryshchep O., Ma-Krupa W., Younge B.R., Goronzy J.J., Weyand C.M. (2008). Vessel-specific Toll-like receptor profiles in human medium and large arteries. Circulation.

[128] Grond-Ginsbach C., de Freitas G.R., Campos C.R., Thie A., Caso V., Machetanz J., Kloss M. (2012). Familial occurrence of cervical artery dissection—Coincidence or sign of familial predisposition?. Cerebrovasc. Dis..

[129] Grond-Ginsbach C., Brandt T., Kloss M., Aksay S.S., Lyrer P., Traenka C., Erhart P., Martin J.J., Altintas A., Siva A. (2017). Next generation sequencing analysis of patients with familial cervical artery dissection. Eur. Stroke J..

[130] Brandt T., Kloss M., Lindner A., Erhart P., Grond-Ginsbach C., Engelter S.T. (2018). Cervical artery dissection in two monozygotic twin-pairs. Eur. J. Neurol..

[131] Traenka C., Kloss M., Strom T., Lyrer P., Brandt T., Bonati L.H., Grond-Ginsbach C., Engelter S. (2019). Rare genetic variants in patients with cervical artery dissection. Eur. Stroke J..

[132] Grond-Ginsbach C., Chen B., Krawczak M., Pjontek R., Ginsbach P., Jiang Y., Abboud S., Arnold M.L., Bersano A., Brandt T. (2017). Genetic Imbalance in Patients with Cervical Artery Dissection. Curr. Genom..

[133] Debette S., Markus H.S. (2009). The genetics of cervical artery dissection: A systematic review. Stroke.

[134] Markus H.S., Levi C., King A., Madigan J., Norris J., Cervical Artery Dissection in Stroke Study I. (2019). Antiplatelet Therapy vs Anticoagulation Therapy in Cervical Artery Dissection: The Cervical Artery Dissection in Stroke Study (CADISS) Randomized Clinical Trial Final Results. JAMA Neurol..

[135] Debette S., Mazighi M., Bijlenga P., Pezzini A., Koga M., Bersano A., Korv J., Haemmerli J., Canavero I., Tekiela P. (2021). ESO guideline for the management of extracranial and intracranial artery dissection. Eur. Stroke J..

[136] Lin J., Sun Y., Zhao S., Xu J., Zhao C. (2016). Safety and Efficacy of Thrombolysis in Cervical Artery Dissection-Related Ischemic Stroke: A Meta-Analysis of Observational Studies. Cerebrovasc. Dis..

[137] Engelter S.T., Lyrer P., Traenka C. (2021). Cervical and intracranial artery dissections. Ther. Adv. Neurol. Disord..

[138] Diana F., Frauenfelder G., Saponiero R., Iaconetta G., Romano D.G. (2022). Endovascular Flow Diversion in Cervical Internal Carotid Artery Dissections. World Neurosurg..

[139] Stella N., Palombo G., Filippi F., Fantozzi C., Taurino M. (2010). Endovascular treatment of common carotid artery dissection via the superficial temporal artery. J. Endovasc. Ther..

[140] Keser Z., Meschia J.F., Lanzino G. (2022). Craniocervical Artery Dissections: A Concise Review for Clinicians. Mayo Clin. Proc..

[141] Brandt T., Caplan L. (2001). Spontaneous Arterial Dissection. Curr. Treat. Options Neurol..

