# Supplementary figures and images for: Unraveling the Links between Chronic Inflammation, Autoimmunity, and Spontaneous Cervicocranial Arterial Dissection
Source: J Clin Med. 2023 Aug 5;12(15):5132. doi: 10.3390/jcm12155132 (PMC10419694; doi:10.3390/jcm12155132)

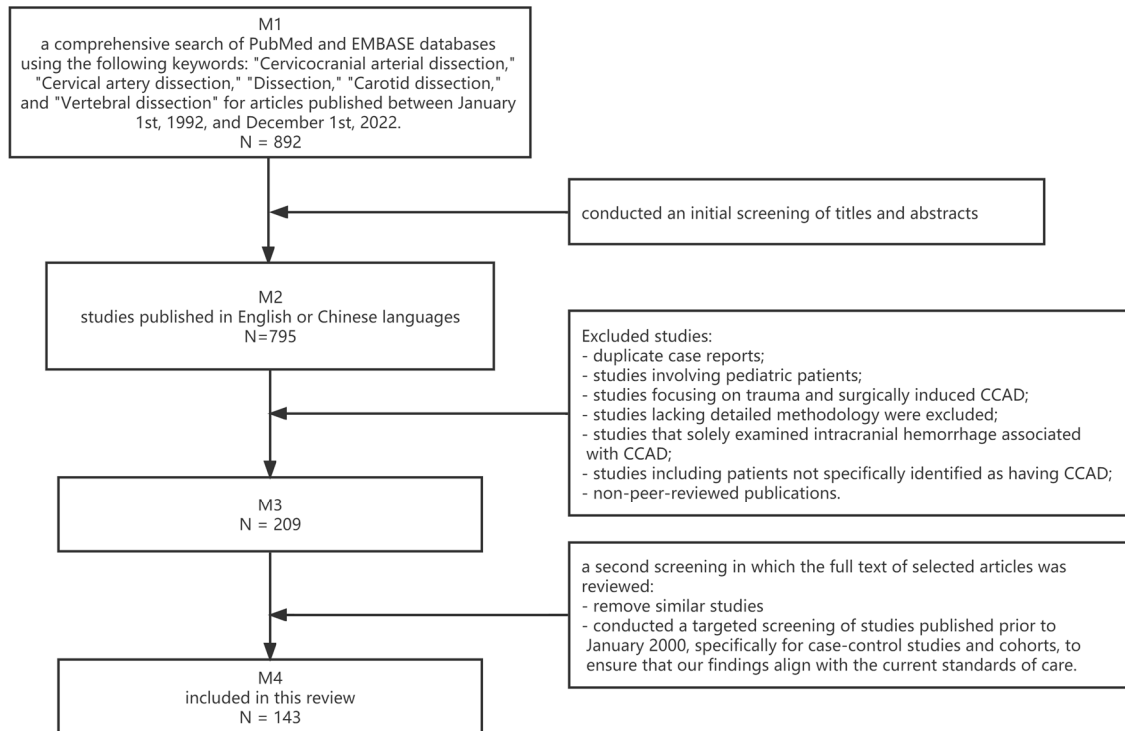

Supplement: Supplementary file 1 [file jcm-12-05132-s001.zip › jcm-2370313-supplementary.pdf]
